# Body mapping of regional sweat distribution in young and older males

**DOI:** 10.1007/s00421-020-04503-5

**Published:** 2020-09-29

**Authors:** Nicole A. Coull, Anna M. West, Simon G. Hodder, Patrick Wheeler, George Havenith

**Affiliations:** 1 Environmental Ergonomics Research Centre, Loughborough School of Design and Creative Arts, Loughborough University, Loughborough, LE11 3TU UK; 2 Diabetes Research Centre, Leicester General Hospital, University of Leicester, Leicester, LE5 4PW UK; 3 School of Sport, Exercise and Health Sciences, Loughborough University, Loughborough, LE11 3TU UK

**Keywords:** Sweating, Skin temperature, Body mapping, Exercise, Heat, Ageing

## Abstract

**Purpose:**

Given the pressing impact of global warming and its detrimental effect on the health of older populations, understanding age-related changes in thermoregulatory function is essential. Age differences in regional sweat distribution have been observed previously, but given the typically small measurement areas assessed, the development of whole body sweat maps for older individuals is required. Therefore, this study investigated age-related differences in regional sweat distribution in a hot environment (32 °C/50%RH) in young and older adults, using a body mapping approach.

**Methods:**

Technical absorbent pads were applied to the skin of 14 young (age 24 ± 2 years) and 14 older (68 ± 5 years) males to measure regional sweat rate (RSR) at rest (30 min) and during exercise (30 min), at a fixed heat production (200 W m^−2^). Gastrointestinal (*T*_gi_) and skin temperature (*T*_sk_), heart rate, thermal sensation, and thermal comfort were also measured.

**Results:**

Whole body sweat maps showed that despite equal heat production, healthy older males had significantly lower gross sweat loss (GSL) than the young and significantly lower RSR at almost all body regions at rest and at the hands, legs, ankles, and feet during exercise. The lower sweat loss in the older group coincided with a greater increase in *T*_gi_ and a consistently higher *T*_sk_ at the legs, despite subjectively feeling slightly cooler than younger individuals.

**Conclusion:**

These findings support the evidence of age-related deterioration in both autonomic and subjective responses in the heat and highlight the lower extremities as the most affected body region.

**Electronic supplementary material:**

The online version of this article (10.1007/s00421-020-04503-5) contains supplementary material, which is available to authorized users.

## Introduction

Under thermoneutral, resting conditions, heat balance is subtly maintained via vasomotor adjustments, whereby heat is dissipated at a similar rate to heat production (Parsons 2014). During exercise and exposure to high environmental temperatures, the challenge of maintaining heat balance is greater, as the requirement for heat dissipation becomes essential to prevent overheating (Smith and Johnson 2016). Under these conditions, evaporation of sweat becomes the primary avenue of heat loss from the body, triggered by an increase in both core temperature (*T*_core_) and skin temperature (*T*_sk_). Thus, understanding the mechanisms that underpin and alter the human sweat response is important to maintain human function.

Human ageing is associated with an alteration in thermoregulatory function during both passive and exercise-induced hyperthermia (Kenney et al. 2014). This age-related alteration includes attenuation of the eccrine sweating response, which consequently impairs heat loss both in warm environmental conditions and during exercise. A reduced sweat response is suggested to be due to a decrease in thermal sensitivity and atrophy of eccrine sweat glands, resulting in a lower sweat output per gland (Inoue and Shibasaki 1996; Kenney and Munce 2003; Smith et al. 2013a). Beginning with a decline in cutaneous vasodilation, the initial reduction in sweat gland output is subsequently followed by a decrease in the number of heat-activated sweat glands (Smith et al. 2013a). These age-related changes in thermoregulatory function may put older individuals at increased risk of hyperthermia-related illness during exposure to warm or hot environments (Kenney and Munce 2003).

As the sweating response plays such a major role in human survival and function, it is not surprising that a large body of research already exists in this area. Previous research has focused on regional (Havenith et al. 2008; Machado-Moreira et al. 2008a; Machado-Moreira et al. 2008b; Smith and Havenith 2011; Smith and Havenith 2012; Smith et al. 2013a; Taylor and Machado-Moreira 2013; Smith et al. 2013b; West et al. 2019) and age-related (Kenney and Anderson 1988; Tankersley et al. 1991; Inoue et al. 1995, 1999a, b; Dufour and Candas 2007; Shibasaki et al. 2013; Smith et al. 2013a) differences in sweat rate. It is evident that variations in regional sweat rates (RSR) exist, with the highest RSR observed on the upper back and forehead and the lowest on the hands and feet (Havenith et al. 2008; Smith and Havenith 2011, 2012). However, despite some research investigating the effect of ageing on the sweating response, typically measuring only a few small areas (2–13 cm^2^—most commonly the thigh, chest, back and arm) (Kenney and Anderson 1988; Tankersley et al. 1991; Inoue et al. 1995, 1999a, b; Dufour and Candas 2007; Shibasaki et al. 2013; Smith et al. 2013a), it is still unknown whether the RSR pattern remains the same in older individuals, across the whole body. Further research in this area is required, now more than ever, due to the rapidly changing climate and pressing impact of global warming on the lives of the older population.

Within the literature, there are several sweat collection techniques and analyses, as well as different methodologies to induce a sweat response. The most common method to assess RSR is the utilisation of ventilated capsules which attach to the skin and continuously measure the difference in vapour content between influent and effluent air (Nadel et al. 1971; Morris et al. 2013). Despite the frequent use of this technique, it is not suitable for the measurement of large areas of skin or whole body regions. More recently, studies have used highly absorbent material placed on the skin to map RSR over the torso (Havenith et al. 2008; Smith and Havenith 2019) and whole body (Smith and Havenith 2011, 2012). This method enables large areas to be measured simultaneously, whereby whole body sweat maps can be created and compared in different population groups.

Comparisons between individuals of different sex, age, or disease state can become complex due to several factors that may influence the sweat response (Cramer and Jay 2014). Many studies have investigated age-related changes during passive heating (Inoue et al. 1991, 1995; Inoue and Shibasaki 1996; Dufour and Candas 2007; Smith et al. 2013a) as this limits some confounding factors; however, some studies have attempted to compare sweat rates during exercise (Anderson and Kenney 1987; Kenney and Anderson 1988; Smolander et al. 1990; Buono et al. 1991; Tankersley et al. 1991; Havenith et al. 1995; Inoue et al. 1999a). The aforementioned studies using exercise as a stimulus for sweating typically prescribe an exercise intensity based on an individual’s %*V*O_2max_. However, according to Cramer and Jay (2014), selecting an intensity to match the individual’s heat production is the most appropriate way to compare independent groups to avoid systematic differences in *T*_core_ and RSR.

Currently, there are limited data assessing age-related differences in the regional distribution of sweat over the whole body during both rest and exercise. Therefore, the aim of this study was to compare RSR in young and older males, using a technical absorbent body mapping approach, during passive and exercise-induced heat strain, at a fixed rate of heat production. It was hypothesised that the older group would have a significantly lower gross sweat loss (GSL) and RSR, and a different RSR distribution compared to the young.

## Methodology

### Participants

Twenty-eight healthy, physically active, white Western European males were recruited for this study from two age ranges: 14 young (mean ± SD: age 24 ± 2 years, height 180.6 ± 7.7 cm, body mass 78.0 ± 11.4 kg, body fat 15.2 ± 3.0%, BSA 2.0 ± 0.2 m^2^, predicted *V*O_2max_ 48.1 ± 6.8 ml kg min^−1^) and 14 older (mean ± SD: age 68 ± 5 years, height 174.9 ± 4.6 cm, body mass 76.4 ± 8.6 kg, body fat 20.9 ± 4.0%, BSA 1.9 ± 0.1 m^2^, predicted *V*O_2max_ 35.5 ± 5.3 ml kg min^−1^). All individuals were recreationally active and free from illness and injury.

Prior to taking part, participants were provided with detailed information about the study and subsequently provided written informed consent. Additionally, all participants were required to complete a health-screen questionnaire and were excluded from the study if they failed to meet the required health standards. Due to the nature of the study, only participants that were non-smokers and had no history of cardiovascular disease, skin/sweat-related conditions or neuromuscular disorders were recruited. All participants in the older age group were required to undergo medical screening carried out by a clinician before taking part in the study and were not taking any medication that could have influenced the results of the study (57% of participants were not taking any medication; however, the most commonly reported medication within this group was for treatment of raised blood pressure and cholesterol). All protocols and procedures were approved by the Loughborough University Ethics Committee (project reference: R16-P134) and are in line with the World Medical Association Declaration of Helsinki for medical research using human participants.

### Experimental design

Participants attended the laboratory on three separate occasions (four visits for older participants including the medical screening visit) which included a pre-experimental/familiarisation session (visit 1) and two main experimental trials (visit 2 and 3). The two experimental trials (UPPER and LOWER) were completed in a balanced order after the pre-experimental session (at least 48 h between each session). All three sessions were completed at the same time of day (± 2 h) for each participant to minimise the influence of circadian rhythm. All sessions were conducted in the Environmental Ergonomics Research Centre at Loughborough University in a climate-controlled environmental chamber (T.I.S.S. Peak Performance, Series 2009 Climate Chambers) in 20 °C/50% RH for the pre-experimental session and 32 °C/50% RH for the main trials.

### Pre-experimental session

The pre-experimental session involved a submaximal exercise test, collection of anthropometric measurements, and familiarisation with the experimental protocol. All participants refrained from alcohol and vigorous exercise 24 h before their visit to the laboratory. On the day of the trial, participants were advised to drink plenty of water (500 ml 2 h prior) to ensure that they were sufficiently hydrated (Casa et al. 2000; Sawka et al. 2007).

#### Anthropometric measurements

On arrival at the laboratory, measures of participant’s height in cm (Stadiometer, SECA, Leicester, UK) body mass in kg (Metter Toledo kcc150, Metter Toledo, Leicester, UK, Resolution 1 g) and body fat percentage via bio-electrical impedance (Body composition analyser, Tanita, MC-780MA) were recorded. Bio-electrical impedance was used due to the simplicity of the measure and its wide use within the literature. Anthropometric measurements were also taken to calculate the size of each individual sweat pad for all participants. The body dimensions and anatomical landmarks used in the production of the sweat pads are in accordance with Smith and Havenith (2011) which were previously modified from the guidelines provided by Lohmann et al. (1988). The same anatomical tape measure was used by the same investigator for all body measurements and values were recorded to the nearest 0.1 cm.

#### Submaximal exercise test

All participants completed a submaximal exercise test on a treadmill (HP Cosmos Mercury 4.0, HP Cosmos Sports & Medical GMBH, Nussdorf-Traunstein, Germany) in a controlled climate (20 °C/50% RH) using an online breath-by-breath analysis system (Quark CPET, COSMED, Rome, Italy). Throughout the test, heart rate was monitored (COSMED, Rome, Italy), and at the end of each stage, participants rated their level of perceived exertion (RPE) using a perceptual scale (Borg 1982). The test was terminated once the participants’ heart rate reached 85% of their age predicted maximum (220-age) or they voluntarily stopped the test. Predicted *V*O_2max_ was determined by extrapolating the heart rate–*V*O_2_ relation to the age predicted maximum heart rate.

#### Metabolic heat production calculation

To prescribe the exercise intensity for the main trials, the rate of metabolic energy expenditure (*M*) and heat production was estimated for each participant using the data collected during the submaximal exercise test. For the purpose of the study, all participants were required to work at a metabolic heat production of 200 W m^−2^ which was estimated as follows (Cramer and Jay 2014):

1 $$M = V{\text{O}}_{{\begin{array}{*{20}c} {2 } \\ \\ \end{array} }} \times \frac{{\left[ {\left( {\frac{{{\text{RER}} - 0.7}}{0.3}} \right) \times e_{c} } \right] + \left[ {\left( {\frac{{1.0 - {\text{RER}}}}{0.3}} \right) \times e_{f} } \right]}}{{60 \times {\text{BSA}}}} \times 1000,$$

2 $${\text{Heat production}} = M - W \left( {{\text{W}}\,{\text{m}}^{ - 2} } \right),$$

where RER is respiratory exchange ratio; BSA is body surface area (m^2^); *e*_*c*_ is energy equivalent of carbohydrate (21.13 kJ) per L of O_2_ consumed (L min^−1^); *e*_*f*_ represent the energy equivalent of fat (19.69 kJ) per L of O_2_ consumed (L min^−1^); heat production was then estimated as the difference between *M* and the external work (*W*) produced (W m^−2^).

### Experimental preparation

#### Gastrointestinal temperature pill

For the measurement of gastrointestinal temperature (*T*_gi_) throughout the experimental trials, each participant swallowed an ingestible temperature pill (VitalSense capsule, Respironics Inc., Germany, Range: 32–42 °C) 5 h before the start of the session and this was monitored using a Vitalsense Integrated Physiological Monitoring System (Mini Mitter Company, Inc. Bend, Oregon, USA).

#### Absorbent sweat pads

Prior to all experimental trials, absorbent sweat pads (Technical Absorbent, Product 2724) were individually cut for each participant based on the measurements taken in the pre-experimental session. The size and positioning of the pads were developed based on the work conducted by Smith and Havenith (2011). A total of 92 pads (46 for rest and 46 for exercise) were sized and produced per participant. Absorbent pads were applied to the torso, arms, and legs, and 100% cotton gloves and socks were applied to the hands (Cotton knit material stitched gloves, The Healthy house Ltd, Stroud, Glos, UK) and feet (Universal Textiles, Leicester, UK), respectively. The location of each pad was identical to previous work (Smith and Havenith 2011), aside from the buttocks, which were not measured in the current study due to the seated rest period and the hands and feet which were measured as one region for speed of application and removal.

All pads were attached to customised plastic sheeting using both single- and double-sided tape to enable rapid application and removal during the measurement periods. Over the top of the plastic sheeting, participants wore a stretch zip long sleeved t-shirt and running leggings (Kalenji, Decathlon) to ensure that all pads were uniformly pressed against the skin and held in position for the duration of the sweat collection period. The ankle pads were held in place inside the cotton socks and cotton gloves were worn on the hands. The cotton gloves were covered with latex laboratory gloves and socks covered with plastic foot covers, creating an impermeable layer to prevent the evaporation of sweat. This also ensured that the cotton was pressed against the skin during the collection period.

To avoid changing the thermal state of the body with the application of the absorbent pads, the experiment was split into two identical trials, as covering the entire body would potentially limit heat dissipation. One trial included sweat collection of the torso, arms, and hands (UPPER), and the other trial included the legs and feet (LOWER).

For the calculation of RSR, each absorbent pad or cotton item (gloves and socks) and an empty airtight zip-lock plastic bag (corresponding to each pad/cotton item and individually labelled) were weighed before and after application, using electronic scales, to the nearest 0.001 g (Kern and Sohn GmbH, D-72336 Balingen, Germany). After application, each pad/cotton item was removed from the plastic sheeting and immediately placed in its corresponding airtight plastic bag (to prevent the evaporation of sweat from the pad) and weighed again. The pre-weight of the absorbent pad or cotton item and plastic bag were subtracted from the post-weight for the calculation of local sweat rate.

### Experimental protocol

All participants refrained from alcohol, vigorous exercise, and application of any sprays or moisturisers 24 h before their visit to the laboratory. On the day of the trial, participants were advised to drink plenty of water (500 ml 2 h prior) to ensure that they were sufficiently hydrated (Casa et al. 2000; Sawka et al. 2007) before exercising in a hot environment.

On arrival at the laboratory, participants changed into standardised clothing, which consisted of a test t-shirt and running shorts provided by the researcher and their own personal trainers and socks for all trials. They then remained in a preparation room for a 30 min stabilisation period where they were fitted with a wrist worn heart rate monitor (Polar A360, Polar Electro Oy, Professorintie 5, FI-90440 Kempele, Finland) and briefed on the experimental process and the use of subjective scales. Participants also provided a urine sample for the assessment of hydration status using a Refractometer (Clinical Master Refractometer, Atago, Japan) and were deemed to be euhydrated if urine specific gravity was < 1.020 (Casa et al. 2000; Sawka et al. 2007).

**Fig. 1:**
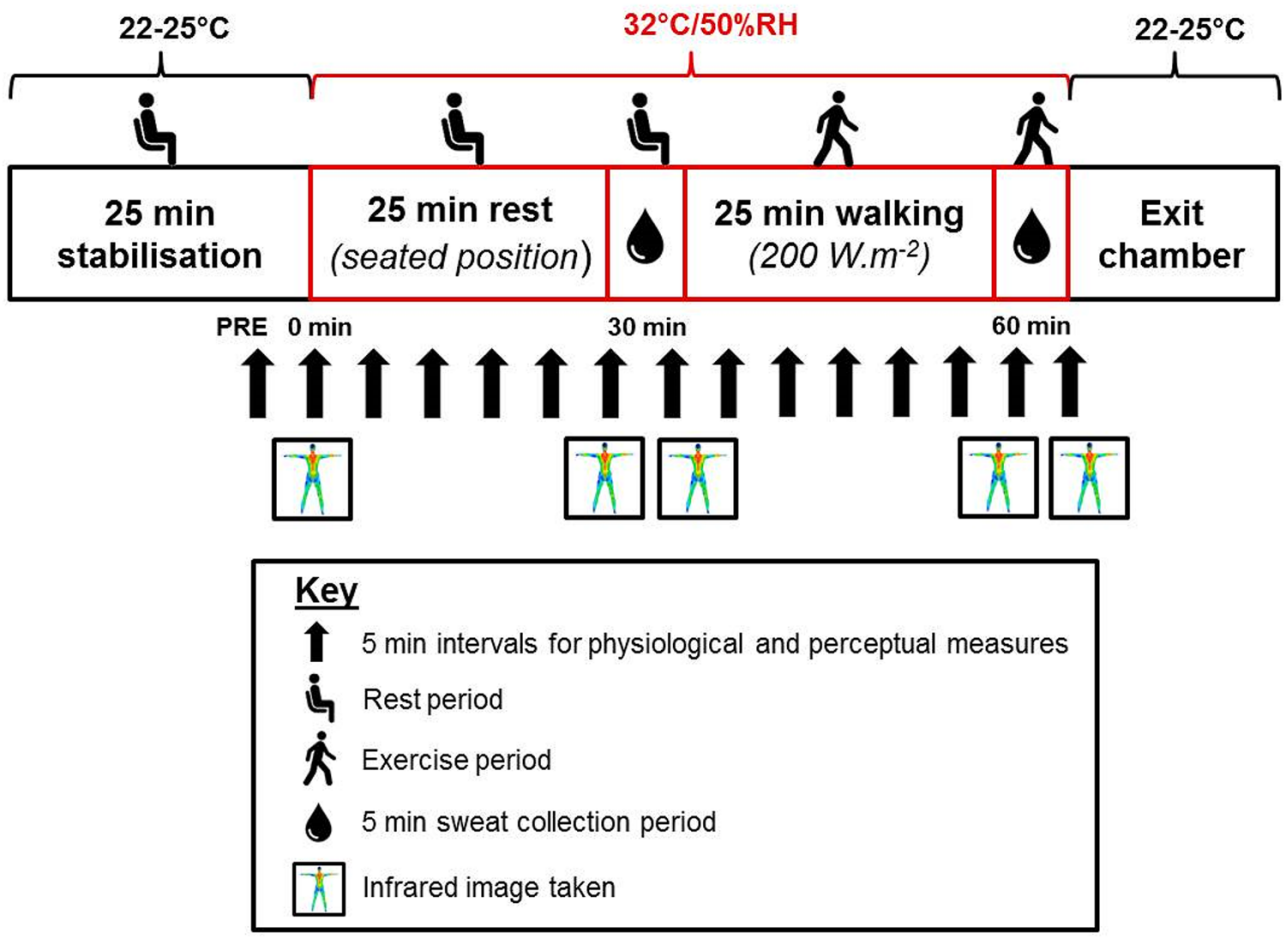
Schematic of the experimental protocol timeline

Once stabilised to a thermoneutral environment, participants entered the environmental chamber (32 °C/50% RH). Participants were not permitted to consume any fluid after entering the chamber until the experimental protocol was complete. Immediately on entering the chamber, participants were weighed semi-nude and then stood in an anatomical position for a thermal image to be taken of their front and back using an infrared camera (FLIR T620, FLIR Systems Inc. Wilsonville, USA). Infrared images were taken throughout the trial (before and after pad application as shown in Fig. 1) to determine mean and regional *T*_sk_ to ensure that the absorbent pads did not change the thermal state of the body during application. Before all images were taken, the participant’s skin was dried down with a towel to remove sweat from the surface.

Participants then rested for 25 min in a perforated, hard plastic chair and every 5 min were asked to rate their whole body thermal sensation (− 50 extremely cold to 50 extremely hot) and thermal comfort (1 comfortable to 7 very uncomfortable) using custom scales, based on previous work (BS EN ISO 10551 2019). During this time, heart rate and *T*_gi_ were also recorded at 5 min intervals as well as environmental temperature and RH (Testo 435-2 with integrated hot wire probe, Testo Ltd, Alton, Hampshire, UK). After the rest period, an infrared image was taken and the absorbent pads were applied to the skin with accompanying stretch zip t-shirt/leggings worn over the top for a total of 5 min for sweat collection at rest (30 min of rest in total). Once the pads were applied to the skin, participants sat back in the chair for the duration of the sweat collection period. Pads were then removed from the skin and the plastic sheeting, and placed into their individual plastic zip-lock bags for weighing as quickly as possible. Participants were towel dried and another infrared thermal image was taken before the post-rest weight (semi-nude) was recorded.

The second part of the protocol involved a 25 min exercise period where participants walked at a fixed heat production of 200 W m^−2^ (3.5 km h^−1^ with varying inclines between participants) on a treadmill (HP Cosmos Mercury 4.0, HP Cosmos Sports & Medical GMBH, Nussdorf-Traunstein, Germany) with a wind speed of 1.5 m s^−1^. During exercise, ratings of thermal sensation, thermal comfort, and RPE (Borg 1982) were recorded alongside heart rate and *T*_gi_. After 25 min of the exercise period, participants dismounted the treadmill for an infrared image and the second application of absorbent pads and began walking again at the same intensity for the 5 min sweat collection period (30 min of exercise in total). Pads were then removed and bagged and the final infrared image and weight recording was taken. Participants then exited the chamber into a thermoneutral environment and remained in the laboratory until *T*_gi_ returned to baseline. The experimental protocol timeline is illustrated in Fig. 1.

#### Infrared images

Throughout the study (time points shown in Fig. 1), whole body infrared thermal images were taken of the participant’s front and back to assess regional and whole body *T*_sk_ as explained previously. The infrared camera was aimed at a perpendicular angle, 2 m away from the body. The camera was calibrated using a blackbody (BLACKPOINT, Blackbody Calibrator, BB702, Omega, USA) which was visible in each infrared image. After each trial, the images were analysed using FLIR Systems Inc. Software (Camera updater and report generator version 2.0 and Thermocam Researcher version 2.8) by selecting regions of interest based on anatomical land marks on the right side of the body only. Whole body *T*_sk_ maps were produced using MATLAB 7.8 software (MATLAB R2013a, The Mathworks Inc, Natick, USA) for a visual representation of the data (only *T*_sk_ maps for the rest period are presented within this paper). For specific image processing details, see Fournet et al. (2013) and Coull (2019).

### Analyses

Calculations to estimate GSL and RSR are detailed below. All statistical analysis is also described in this section.

### Gross sweat loss

The total amount of sweat lost (GSL) was calculated based on the weight change of each participant during the rest and exercise period of each trial, after being towel dried. Adjustments were made for respiratory and metabolic mass losses in line with Smith and Havenith (2011).

### Regional sweat rate

RSR were calculated from the change in weight of each absorbent pad, the surface area of each pad, and the duration of the application to the skin. Control samples of the materials used in the sweat collection (pads, socks, and gloves) were produced to determine the dry weight per unit area, which was then used in the calculation of surface area of each region.

The data from the UPPER and LOWER trials in the rest and exercise period were combined to create whole body sweat maps. As the GSL in the two trials were slightly different (daily variation in participant; differences in covered areas), a correction was applied to standardise RSR for this variation (Smith and Havenith 2011). This correction is based on the assumption that for a specific condition, there was a relation between RSR and GSL. Using the ratios of both sessions’ GSL to the overall mean GSL, RSR data were adjusted to accommodate this issue.

The mean, median, and standard deviation were calculated for all RSRs, for use within the analysis. For the purpose of this study, whole body sweat maps were created using median RSR to show the ‘average sweater’ rather than the average amount of sweat produced, in line with Smith and Havenith (2011). RSR was also compared between the right and left sides of the body for the rest and exercise period in both age groups.

### Statistical analysis

Statistical analysis was completed using Microsoft Excel and SPSS statistical software package (SPSS version 23.0, IBM, USA). Differences in RSR and *T*_sk_ between age groups were assessed using a two-way ANOVA (age and region). The large number of comparisons may increase the likelihood of inflating type I error, and so, Bonferroni corrections were applied to account for multiple comparisons. However, correcting the data using such a conservative correction factor decreases the significance *p* value, thus increasing the risk of type II error (Havenith et al. 2008). For these reasons, both Bonferroni corrected and uncorrected data are presented within the results for the reader to evaluate. Objective and subjective data, including GSL, hydration status, *T*_sk_, *T*_gi_, thermal sensation, thermal comfort, and RPE, were analysed using independent samples *t* tests (two-tailed). Pearson correlations were performed to assess relationships between *T*_sk_, fitness level, and RSR. Unless otherwise stated, data are mean ± SD and significance was set at the *p* < 0.05 level.

## Results

All physiological and subjective responses were compared between UPPER and LOWER trials in both age groups and no significant differences (excluding GSL) were observed (*p* > 0.05). It was, therefore, deemed appropriate to combine data from both trials to compare between age groups. Unless otherwise stated, the data presented below is an average of the UPPER and LOWER trials over the duration of the protocol.

### Participant characteristics

Independent samples *t* tests confirmed significant differences in age (young: 24 ± 2 years vs. older: 68 ± 5 years, *p* = 0.0001), height (young: 180.6 ± 7.7 cm vs. older: 174.9 ± 4.6 cm, *p* = 0.03), body fat percentage (young: 15.2 ± 3.0% vs. older: 20.9 ± 4.0%, *p* = 0.001), and *V*O_2max_ (young: 48.1 ± 6.8 ml kg min^−1^ vs. older: 35.5 ± 5.3 ml kg min^−1^, *p* = 0.001) between groups. No significant differences (*p* > 0.05) were observed in body mass (young: 78.0 ± 11.4 kg vs. older: 76.4 ± 8.6 kg) or BSA (young: 2.0 ± 0.2 m^2^ vs. older: 1.9 ± 0.1 m^2^) between groups.

### Hydration status

All participants were within the euhydrated range (< 1.020) on arrival to the laboratory. There was no significant difference in hydration status (*p* > 0.05) between the young (1.011 ± 0.006) and older group (1.010 ± 0.008).

### Gastrointestinal temperature

**Fig. 2:**
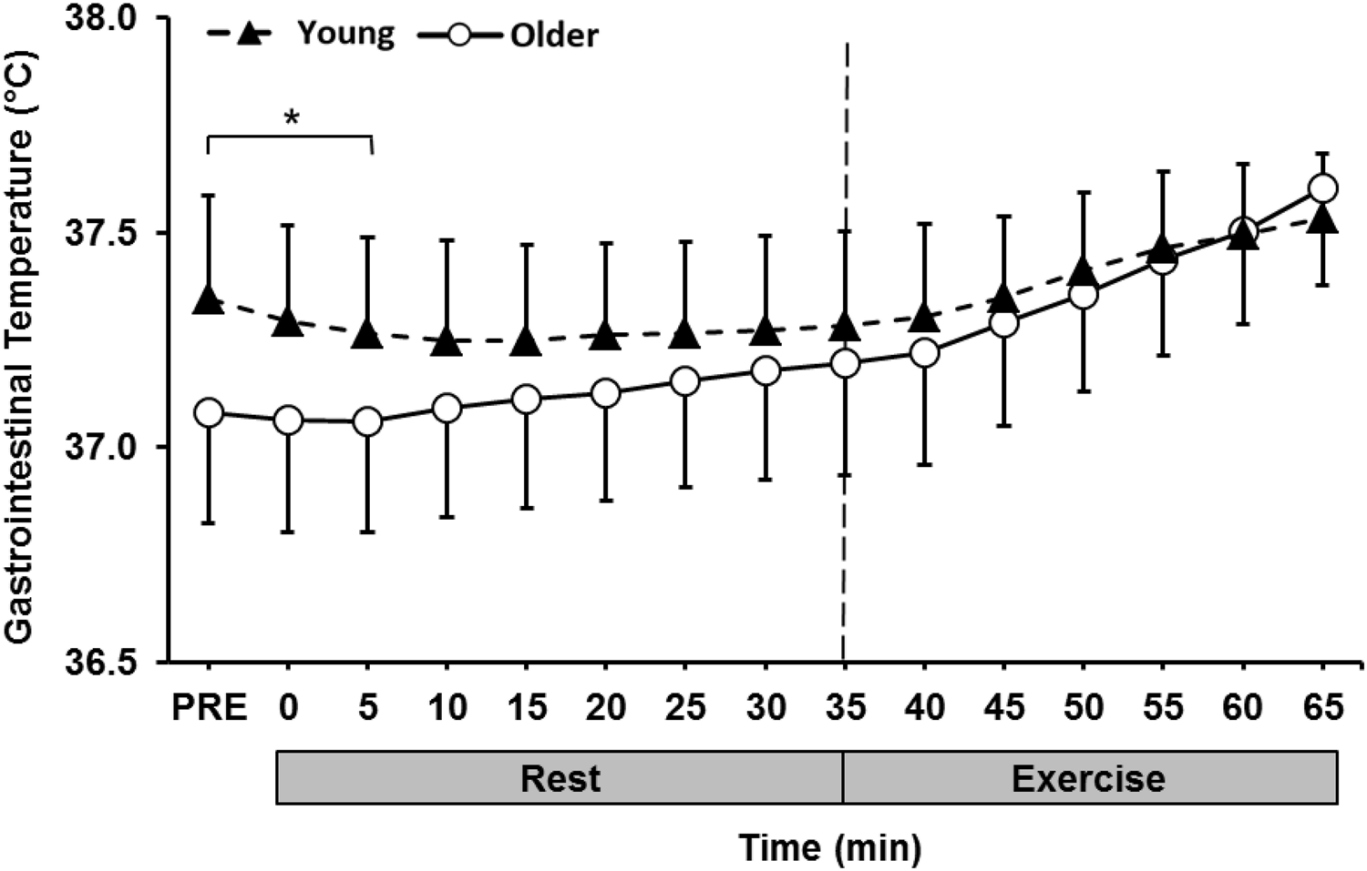
Gastrointestinal temperature (°C) during the rest and exercise period (32 °C and 50% RH) in the young (18–30 years) and older (60–80 years) group. *Significantly different between groups

**Fig. 3:**
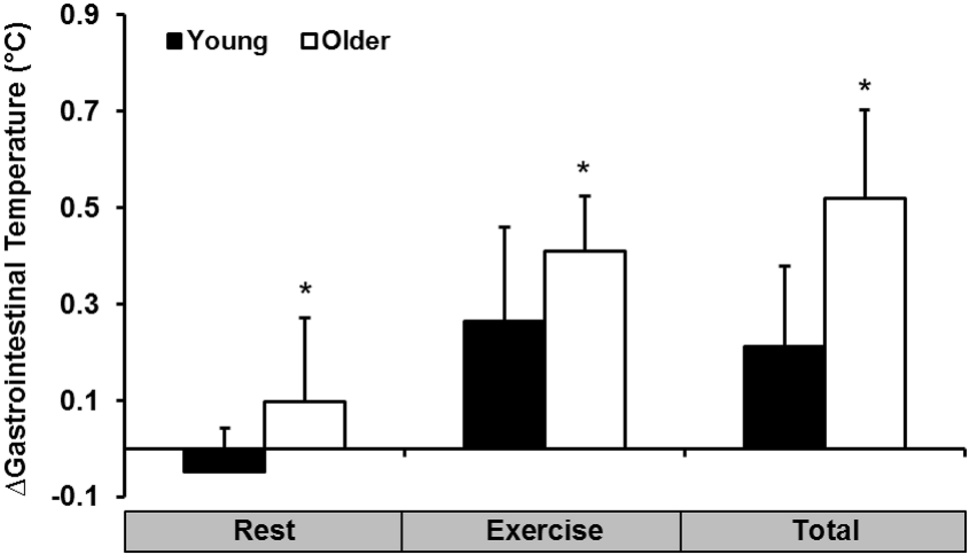
***∆***Gastrointestinal temperature (°C) during the rest and exercise period (32 °C and 50% RH) in the young (18–30 years) and older (60–80 years) group. *Significantly different between groups

Older individuals had a significantly lower *T*_gi_ from PRE to 5 min into the trial (*p* < 0.05) when compared to the young group (Fig. 2). *T*_gi_ remained lower in the older group up until 60 min into the trial where it began to surpass the younger group during the exercise period. The ***∆T***_gi_ from pre-post in the rest and exercise period was compared between age groups (Fig. 3). Older individuals had a significantly higher ***∆T***_gi_ in both the rest (*p* = 0.01) and exercise (*p* = 0.03) period of the trial when compared to the young group.

### Heart rate

During the rest period, heart rate remained stable in both groups before an expected increase at the commencement of exercise (shown in ESM1, electronic supplementary material, available online). Older individuals had a significantly lower heart rate from 5–40 min to 50–65 min during the trial when compared to the young group (*p* < 0.05). However, these values represent a significantly higher percentage of heart rate max (220-age) for older individuals at all time points and thus when this is accounted for the graph would display the opposite. On average, percentage heart rate max was 41% at rest and 53% during exercise in the young group and 49% and 64% in older individuals, respectively.

### Thermal sensation and comfort

**Fig. 4:**
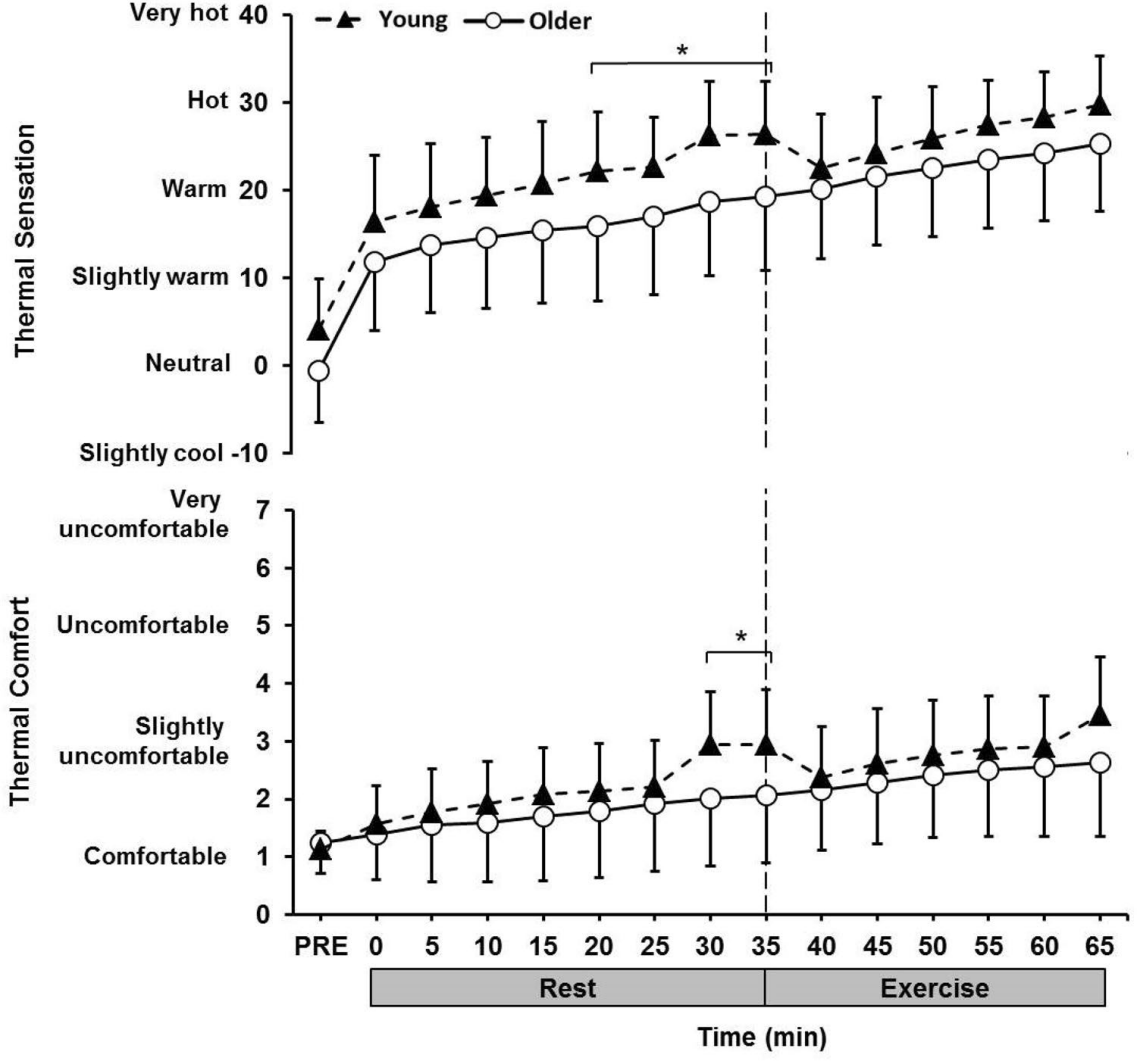
Thermal sensation and thermal comfort responses during the rest and exercise period (32 °C and 50% RH) in the young (18–30 years) and older (60–80 years) group. *Significantly different between groups

There were significant differences in thermal sensation between age groups at PRE and 20–35 min (*p* < 0.05), as shown in Fig. 4. Mean responses in both age groups were between ‘slightly warm’ and ‘slightly cool’ at the beginning of the trial and reached ‘warm’ to ‘hot’ towards the end. A similar trend was observed for thermal comfort between groups, with older individuals’ responses remaining lower throughout the trials (Fig. 4). However, these responses were only significantly lower than the young group between 30 and 35 min (*p* < 0.05). Participants in both groups perceived themselves to be ‘comfortable’ at the beginning of the trial, reaching ‘slightly uncomfortable’ towards the end.

### Rating of perceived exertion

Throughout exercise, RPE was stable in both groups; however, it was significantly higher (*p* < 0.05) in the older age group (10.8 ± 1.6) when compared to young (8.8 ± 1.6), which coincides with heart rate when expressed as % of heart rate max.

### Gross sweat loss

GSL loss in the UPPER and LOWER trials were combined for an overall GSL comparison between age groups. The younger individuals had a significantly higher GSL during rest (*p* = 0.0001) and exercise (*p* = 0.01), as shown in Table. 1.

**Table 1:** Mean gross sweat loss (UPPER and LOWER trials combined) (over 30 min period) and surface area weighted sum of sweat rates of all pads (during the last 5 min) in the young (18–30 years) and older (60–80 years) group

	**Gross sweat loss (GSL) (g)**		**Gross sweat rate (g m**^**−2**^** h**^**−1**^**)**		**Surface area weighted** **Regional sweat rate (RSR) (g m**^**−2**^** h**^**−1**^**)**	
**Group**	**Rest**	**Exercise**	**Rest**	**Exercise**	**Rest**	**Exercise**
Young	102.8 ± 21.6	275.0 ± 49.6	99.3 ± 18.3	259.6 ± 28.6	101.3 ± 31.3	250.5 ± 40.2
Older	51.9 ± 18.8*	229.9 ± 54.2*	49.8 ± 19.6*	216.9 ± 43.7*	57.9 ± 21.1*	215.2 ± 55.7*

*Significantly different between groups (*p* ≤ 0.01)

### Gross sweat loss and VO_2max_

The relationship between total combined GSL and predicted *V*O_2max_ (from submaximal test) for the young and older group was assessed, and a Pearson’s correlation was performed. No relationship was observed between GSL and *V*O_2max_ in the young (*r* = − 0.19, *p* = 0.52) or older groups (*r* = 0.20, *p* = 0.50).

### Regional sweat rate

Differences in RSR between the right and left sides of the body were observed at several body regions (between 11 and 54 g m^−2^ h^−1^). In the older group, the right posterior lower arm had a significantly higher RSR compared to the left during rest (*p* = 0.04) and exercise (*p* = 0.01). In the young group at rest, the right shoulder (*p* = 0.01), side (*p* = 0.01), and lateral upper leg (*p* = 0.02) had significantly higher RSRs than the left side, and during exercise, this was evident at the anterior (*p* = 0.01) and posterior lower arm (*p* = 0.001) and the lateral lower leg (*p* = 0.03). After Bonferroni corrections, right-to-left differences were only evident at the posterior lower arm in the young group in the exercise period. As these differences only represented a small number of the total regions tested, it was decided that grouping right and left RSR was appropriate for analysis between age groups. This also reduced the amount of comparisons to be made between regions and thus decreased the likelihood of Type I error in line with Smith and Havenith (2011, 2012).

**Fig. 5:**
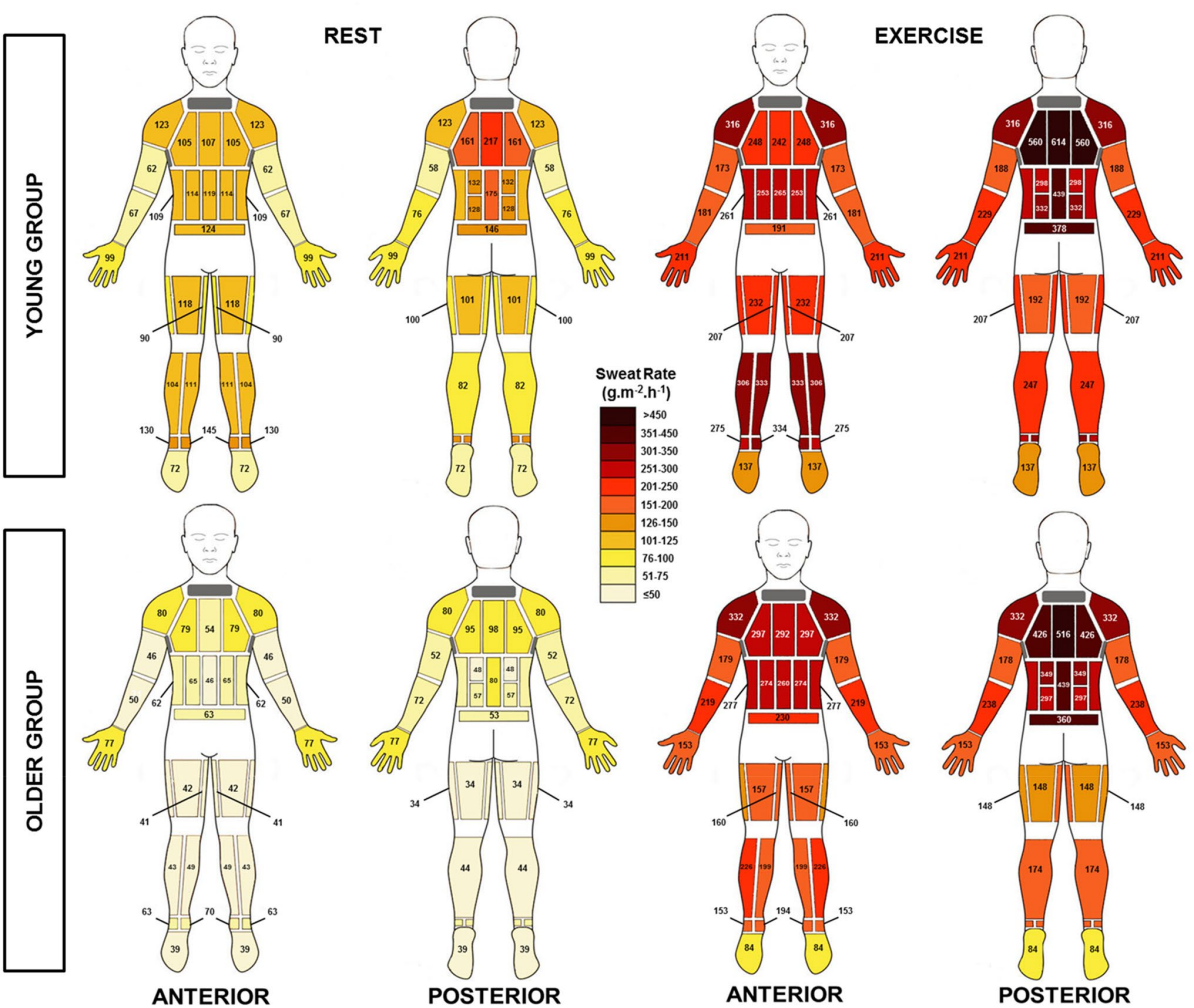
Body maps of absolute regional median sweat rates (g m^−2^ h^−1^) during the rest and exercise period (32 °C and 50% RH) in the young (18–30 years) and older (60–80 years) group

Median grouped data for both age groups are illustrated in the whole body sweat maps for the rest and exercise period (Fig. 5). Descriptive statistics of the RSR for rest and exercise values are presented in electronic supplementary material (ESM2), available online. During passive heating (rest), younger individuals had higher RSR at all body regions; however, these differences were only significant at 20 out of 28 regions, mainly the torso and all regions at the legs and feet (*p* < 0.05). After Bonferroni corrections, younger individuals had significantly higher RSR at lower body regions only (10 regions; legs, ankles, and feet). During the exercise period, young individuals had significantly higher RSR than the older group in 11 out of 28 regions **(**hands (*p* = 0.03) and all lower body regions (*p* ≤ 0.03)). However, after Bonferroni corrections, these differences were only significant at the lateral ankle and the feet (*p* = 0.001). RSR during the exercise period were significantly higher than rest at all body regions in both age groups (*p* ≤ 0.001). The same findings were observed after Bonferroni corrections.

### Normalised regional sweat rate

**Fig. 6:**
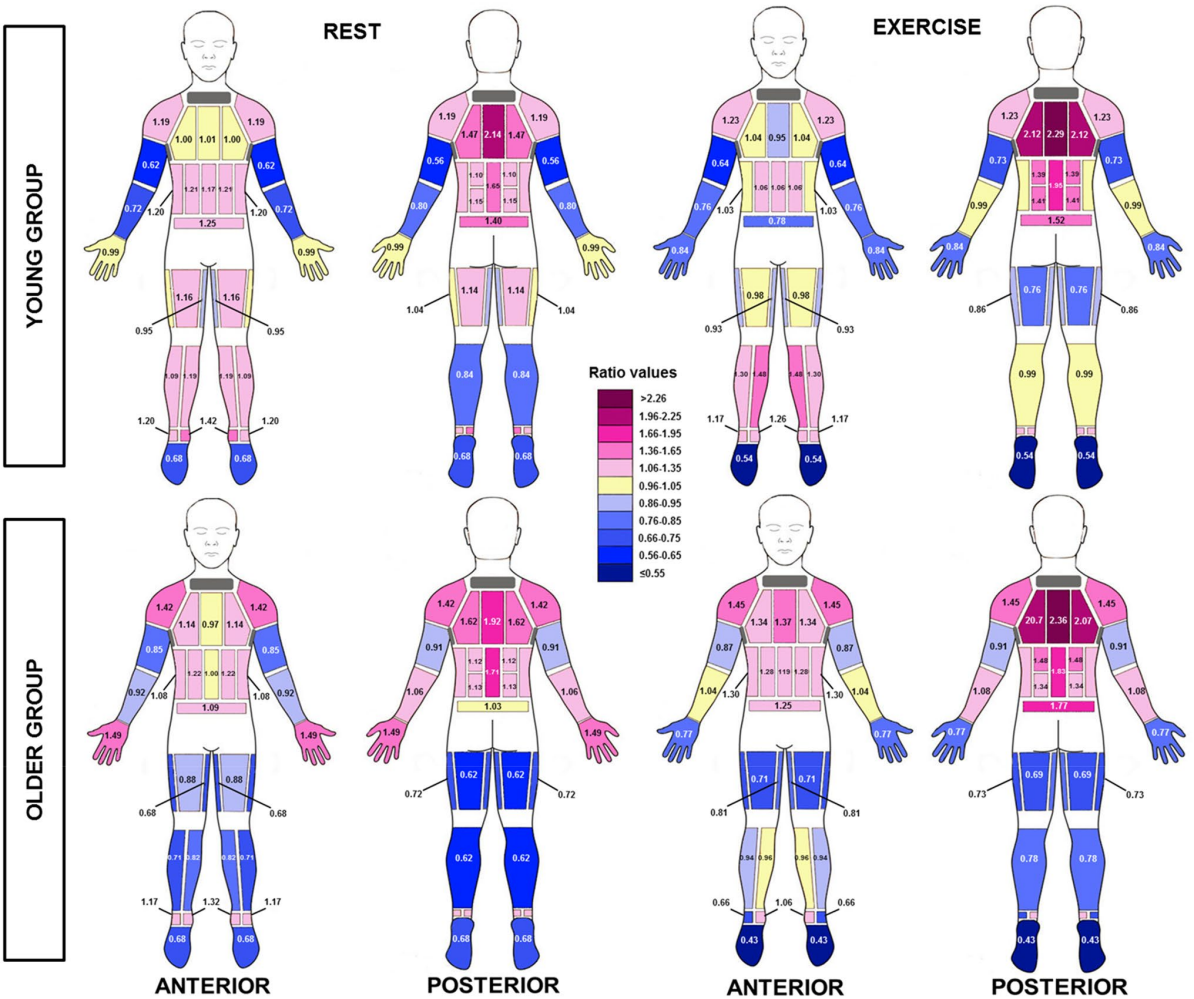
Body maps of normalised regional median sweat rates (RSR) during the rest and exercise period (32 °C and 50% RH) in the young (18–30 years) and older (60–80 years) group. Normalisation was achieved by dividing all local sweat rates by the surface area weighted whole body sweat rate of all zones for each individual. Values > 1 = higher than average sweat rate, 1 = average sweat rate, < 1 = less than average sweat rate

For presentation in Fig. 6, RSR were normalised for the surface area weighted whole body sweat rate of all zones for each individual and then a median of all individuals was used, in line with Smith and Havenith (2011, 2012). The distribution pattern in RSR between rest and exercise was similar with the extremities being lower than the torso. A value of 1 is equal to mean whole body sweat loss, where values of > 1 and < 1 show areas that are lower and higher than whole body sweat loss, respectively.

### Regional skin temperature

Regional *T*_sk_ was compared between age groups at each time point. For regional *T*_sk_ data, please refer to the electronic supplementary material (ESM3), available online. There were several significant differences (torso, legs, and hands) between groups at baseline which may highlight a slight variation in natural temperature distribution due, for example, to blood flow distribution. During the rest and exercise periods in the heat, older individuals had a consistently higher *T*_sk_ at the leg regions (*p* ≤ 0.02) and a lower *T*_sk_ at the posterior upper arm (*p* < 0.05).

**Fig. 7:**
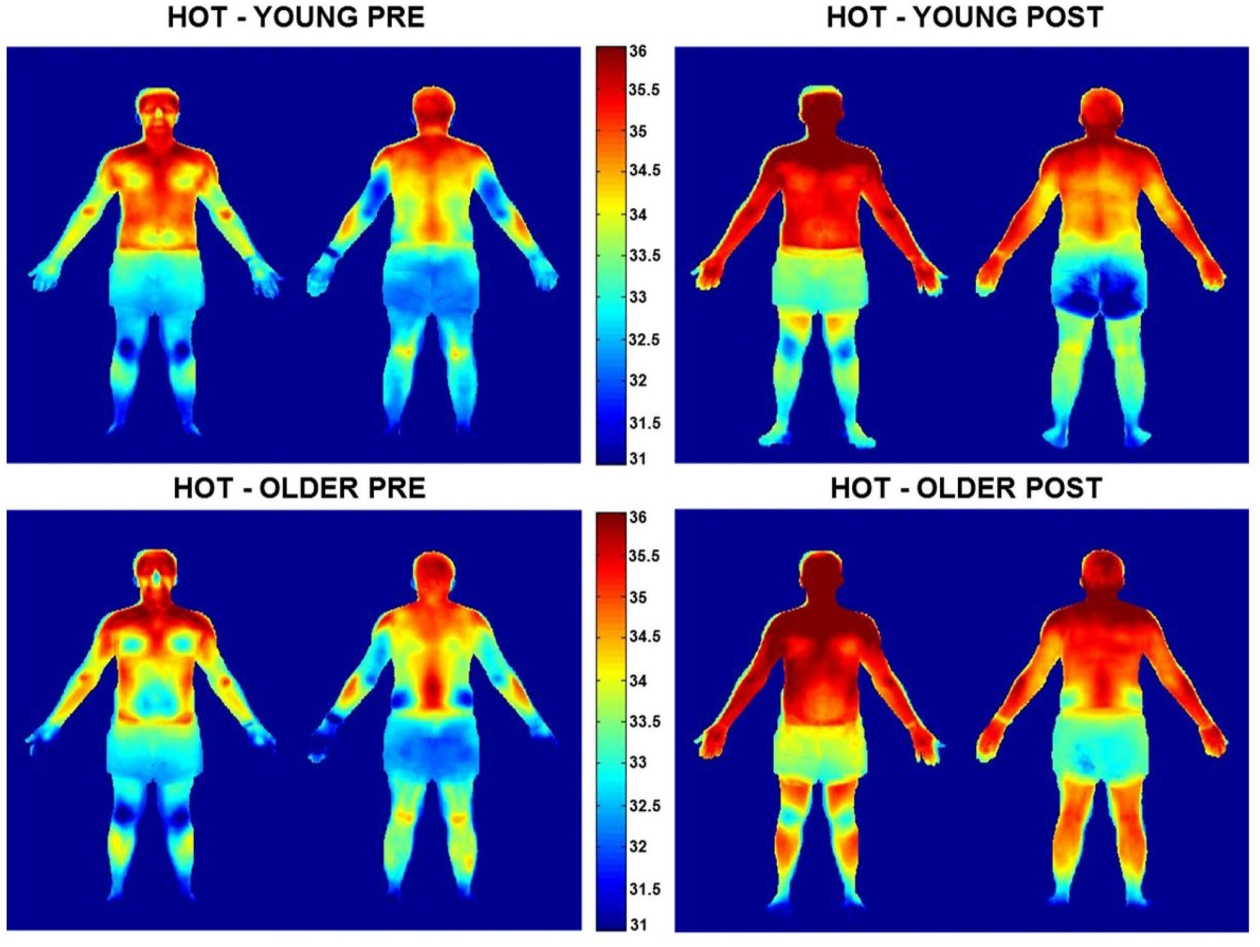
PRE and POST rest period (32 °C and 50% RH), group averaged (*n* = 10 in the young (18–30 years), and *n* = 10 in the older (60–80 years) group) body maps of absolute skin temperature (°C). Temperature scale is adapted based on the overall mean skin temperature of the condition (~ 34 °C) ± 4 °C

Regional *T*_sk_ was also compared between time points [baseline, pre-pad application at rest (pre-pad-rest), post-pad application at rest (post-pad-rest), pre-pad application during exercise (pre-pad-ex), and post-pad application during exercise (post-pad-ex)] within each age group to assess the influence of pad application and exercise (ESM3). At rest, there was a significant increase in *T*_sk_ during the heat exposure at all body regions (*p* < 0.05) in the older group and at all regions except the upper back (*p* = 0.11) in the younger group. During exercise, there was a significant decrease in *T*_sk_ at almost all regions in both age groups (*p* < 0.05) except for the legs, feet, and posterior upper arms showing a significant increase in the young group, as did the legs, hands, and feet in the older group (*p* < 0.05). During the pad application period at rest, there was a small but significant increase in *T*_sk_ (between 0.2 and 0.5 °C) at 6 of 18 body regions in young and 5 of 18 in older individuals (*p* < 0.05). After Bonferroni corrections, this was only significant at 4 body regions. Over the 5 min exercise pad application period, there was a small but significant increase in *T*_sk_ at almost all body regions in the young (15/16 regions) and older group (13/16 regions) (*p* < 0.05). After Bonferroni corrections 11/16 and 12/16 regions were still significantly different in the young and older group, respectively. A visual representation of the skin temperature variation difference between groups is shown in Fig. 7.

Pearson’s correlations were performed to assess the relationship between RSR and local *T*_sk_ at rest (post-pad-rest) and exercise (post-pad-ex) in both age groups. No relationship was observed at any of the body regions assessed (*p* > 0.05) in either age group.

## Discussion

The present study assessed the age-related differences in RSR distribution in young (18–30 years) and older (60–80 years) individuals during rest and exercise in a hot environment (32 ± 0.1 °C/50.5 ± 0.8% RH). Whole body sweat maps were created for both age groups to illustrate the distribution pattern over the body. The main findings were that, although working at the same fixed rate of heat production (200 W m^−2^), older individuals had (1) significantly lower GSL than the young group and (2) significantly lower RSR at almost all body regions during rest and at the hands, legs, ankles, and feet during exercise. The lower RSR observed in the leg region of older individuals coincided with a higher *T*_sk_, indicative of lower evaporative cooling. Furthermore, despite a significantly higher increase in *T*_gi_ than the young group, older individuals felt slightly cooler throughout both the rest and exercise period (particularly during the pad application), suggesting a reduced sensitivity to temperature increase. These findings add to the body of literature that suggests older individuals are at increased risk of heat-induced illness and injury, compared to their younger counterparts, due to a lower GSL, a disproportional reduction in RSR at the lower extremities, a reduced thermal sensitivity, and impaired defence against a rise in *T*_gi_.

### Gross sweat loss

Despite the use of a fixed heat production protocol, substantial inter- and intra-individual variation was observed in GSL in the present study, in agreement with the previous work (Smith and Havenith 2011, 2012). GSL increased significantly from rest to exercise as expected, caused by an increase in metabolic heat production and a proportional increase in sweat loss.

The previous studies have observed a higher GSL in participants with higher *V*O_2max_ values and thus higher fitness levels, when exercising at a fixed load (W) (Havenith et al. 1995) and a %*V*O_2max_ (Havenith et al. 1998; Smith and Havenith 2011, 2012). However in the present study, all participants were exercising at the same fixed heat production (200 W m^−2^) to avoid bias when comparing across age groups (Cramer and Jay 2014), and as expected, no relationship was observed between GSL and predicted *V*O_2max_. This finding supports the conclusions of previous studies which state that GSL does not differ between fit and unfit individuals during exercise at a fixed heat production (Jay et al. 2011; Cramer et al. 2012; Gagnon and Kenny 2012), particularly at rates of 250 W m^−2^ or lower (Gagnon and Kenny 2012). The findings are, however, equally consistent with Smith and Havenith’s observations working at equal %*V*O_2max_ (higher GSL with higher fitness), as they showed an excellent correlation between GSL and metabolic rate. Thus, in a scenario where people work at a %*V*O_2max_ (e.g., while running in a race), given the higher metabolic rates in the fitter (faster) people, they are expected to produce more sweat, as indeed observed.

GSL was significantly lower in the older group compared to the young group during both rest and exercise in the present study. This finding is in agreement with the previous literature during passive heating (Inoue et al. 1995, 1998; Inoue and Shibasaki 1996) and exercise (Anderson and Kenney 1987; Kenney and Anderson 1988), supporting the notion that with increasing age comes a decrease in sweat gland function (Inoue and Shibasaki 1996), which will be discussed in detail below. In the present study, the observed age-related difference in GSL, in the absence of a relationship between GSL and predicted *V*O_2max_, could propose a true age-related change in sweating mechanisms and not fitness level-based differences. However, further research is required to assess GSL during an actual *V*O_2max_ test to confirm this. For the rest period, the observed lower GSL in the older group may be at least partially related to their lower heat production. While resting metabolic rate was not measured in this study, a lower metabolic rate at rest was observed in the older group in the previous work (Coull 2019) with these age groups (older: 43.0 ± 4.6 vs young: 57.1 ± 6.7 W m^−2^; supine).

### Regional sweat rate

This is the first study to provide RSR data over the whole body surface in both young and older individuals (see Fig. 5). From these data, it is clear that there is RSR variation both within and between age groups and body segments, which is in accordance with the previous literature (Kuno 1956; Inoue et al. 1991; Cotter et al. 1995; Inoue and Shibasaki 1996; Havenith et al. 2008; Machado-Moreira et al. 2008a; Smith and Havenith 2011, 2012). Despite this inter-individual variation in RSR, a number of similar patterns were consistently observed.

As expected, RSR increased in parallel with GSL from rest to exercise at all body regions and the distribution patterns remained similar within both age groups.

In the young group, the highest RSR were observed at the posterior torso, followed by the anterior torso, legs/feet then arms/hands. In particular, the upper region of the posterior torso had the highest RSR and the feet had the lowest. This pattern mirrors the findings from previous sweat mapping research in young males (Smith and Havenith 2011), albeit with lower absolute values, due to differences in the exercise intensities employed. Similar distribution patterns have also been observed with studies assessing variation within body segments, including the hand/arms (Smith et al. 2007) and torso (Havenith et al. 2008; Machado-Moreira et al. 2008a) and other studies assessing multiple regions (Cotter et al. 1995; Smith et al. 2013a).

The older group showed a similar RSR pattern for both rest and exercise, with the exception of the extremities. The legs were shown to have a lower RSR than the arms, particularly during exercise. Compared to the younger group, older individuals had significantly lower RSR at several regions of the torso at rest and at all leg regions at both rest and during exercise. A significant body of literature conducted by Inoue and colleagues has consistently observed lower RSR at the leg regions (single sweat capsule sample at thigh) in older individuals (Inoue et al. 1991, 1995, 1998, 1999a, b; Inoue and Shibasaki 1996). However, within some of the aforementioned studies, the decrements were also noted at several other body regions including the back (Inoue 1996; Inoue et al. 1998), chest, and forearm (Inoue et al. 1998). After conducting a substantial amount of research in this specific area, Inoue and Shibasaki (1996) concluded that the age-related decline in heat loss effector function is likely to occur successively in cutaneous vasodilation, followed by sweat output per gland and density of active sweat glands. These decrements are suggested to proceed from the lower extremities, posterior upper body, anterior upper body, and lastly to the head (Inoue and Shibasaki 1996).

Other previous research has aimed to elucidate the exact physiological mechanisms responsible for the age-related differences in sweat rate (Tankersley et al. 1991; Inoue et al. 1999b; Smith et al. 2013a). Some studies have previously postulated that ageing per se has no influence on the sweat response, and instead, the decrement in sweat rate is related to the expected reduction in physical fitness and/or habitual physical activity level (Drinkwater et al. 1982; Smolander et al. 1990; Havenith et al. 1995). However, further studies identified that such declines in sweating still exist when young and older individuals are matched for aerobic fitness and activity levels (Tankersley et al. 1991; Armstrong and Kenney 1993; Inoue et al. 1999a; Smith et al. 2013a). Several factors may contribute to the lower sweat rates observed in older individuals including decreased heat-activated sweat gland function (Anderson and Kenney 1987; Kenney and Anderson 1988; Inoue et al. 1991; Inoue and Shibasaki 1996; Smith et al. 2013a), lower sensitivity to acetylcholine (Kenney and Fowler 1988; Inoue et al. 1999b), and a decreased thermal sensitivity (Natsume et al. 1992).

There is increasing evidence to suggest that a lower sweat gland output, caused by progressive atrophy of the sweat gland itself, is the primary contributing factor of the age-related changes in the sweating response (Sato and Timm 1988; Inoue et al. 1999b, 2004; Kenney and Munce 2003; Shibasaki et al. 2013; Smith et al. 2013a). As discussed above, it may be that this occurs prior to a decrease in heat-activated sweat gland density, as the previous studies have observed similar sweat gland densities across age groups (Anderson and Kenney 1987; Inoue et al. 1991; Inoue and Shibasaki 1996). Although this theory seems to be well documented, there are still questions surrounding the regional pattern of this age-related decline (Smith et al. 2013a). A peripheral-to-central decline has been suggested to be the most logical hypothesis (Kenney and Munce 2003). While we observed higher RSR at the lower extremities than the upper, indeed, it seems that RSR at the lower legs is reduced by 52% more than the upper legs with ageing (difference between age group upper and lower values during exercise period, Figs. 5, 6). As the pattern for the arms is different, the present study, perhaps, provides more support for the differentiating (between extremities) theory of Inoue and colleagues.

To assess RSR over the whole body, it was decided that the use of technical absorbent was the most appropriate method. Although this method is not new to the literature, it is rarely utilised in studies assessing RSR, which is surprising considering that it provides an inexpensive, easy-to-use alternative to the ventilated capsule method (Morris et al. 2013). Ventilated capsules only cover small areas of skin (2–13 cm^2^), which may not be representative of the whole body segment (Havenith et al. 2008). In this context, it should be noted that many of the aforementioned RSR studies were limited to ~ 2 to 6 sweat capsules over the entire body. Consequently, their conclusions drawn upon age-related changes in RSR distribution, specifically over the extremities, are taken from a single small sample from the arm and the leg (typically the thigh). Utilising technical absorbents, the present and earlier data (Smith and Havenith 2011, 2012), show that large RSR variance exists over the extremities, and therefore, we propose that RSR inferences based on a limited number of capsules should be interpreted with caution.

### Regional skin temperature

Regional fluctuation is evident in *T*_sk_ as a result of a number of factors, including alterations in skin blood flow and evaporative cooling of the skin from sweating. Despite this association, there was no significant relationship between the rise in *T*_sk_ and RSR in the present study at rest or during exercise, in agreement with the previous research (Cotter et al. 1995; Smith and Havenith 2011, 2012). Local *T*_sk_ significantly increased during the rest period at all body regions in the older group and all except the upper back in the young group. It is noteworthy that both age groups *T*_sk_ decreased at the majority of regions during exercise, which may be explained by the increase in evaporative cooling and a higher air velocity (1.5 m s^−1^) compared to rest. Despite whole body towel drying to mitigate the build-up of excess sweat, continued evaporative sweat loss during the infrared images may also have contributed to the lower *T*_sk_ observed after exercise.

Age-related differences in regional *T*_sk_ were observed during both rest and exercise in the current study (ESM3). Older individuals had a significantly higher *T*_sk_ at the leg regions when compared to the young, which coincides with the lower RSR observed in this region. Together, this is indicative of a lower evaporative cooling in the older compared to younger group. For visual purposes, Fig. 7 illustrates these age-related differences in *T*_sk_ (at the end of the rest period) in the form of a body map. When combining Fig. 7 with the sweat maps data, it is evident that the legs are the most affected body area in relation to the decline in thermoregulation with age.

As the sweat measurement with absorbent pads involves covering large areas of skin, the impact of *T*_sk_ changes in the sampling period must be considered due to the risk of artificially increasing RSR. At rest, the *T*_sk_ of some regions increased significantly by ~ 0.4 °C while during the exercise sample period, *T*_sk_ increased by up to ~ 0.6 °C. This is a relatively small rise in *T*_sk_ when compared to the average increase observed in the previous studies (Smith and Havenith 2011, 2012), using the same pad application technique (> 1 °C). An increase in *T*_sk_ is arguably unavoidable when utilising this technique even despite the short application periods of the absorbent material (5 min). However, the authors of the aforementioned studies concluded that regional sweat variation could not be explained by observed regional variations in *T*_sk,_ which also holds true for the present findings. In support of this, a recent study underlines the limited role of *T*_sk_ in sweat control (Ravanelli et al. 2020).

### Gastrointestinal temperature, thermal sensation, and comfort

During exposure to the heat, the older individuals had a significantly greater rise in *T*_gi_ during both rest and exercise, when compared to the young group. This was evident despite all participants working at the same fixed rate of heat production. The increased heat strain observed in the older group is a result of a decreased ability to dissipate heat through vasomotor adjustments and sweating and has been observed in numerous previous studies, albeit not implementing fixed heat production protocols (Anderson and Kenney 1987; Sagawa et al. 1988; Inoue et al. 1991; Dufour and Candas 2007; Smith et al. 2013a). The rise in *T*_gi_ observed does not seem threatening under controlled laboratory conditions; however, many older individuals typically spend longer durations exposed to heat stress than in this study, especially during the summer months, and thus are at increased risk of heat-induced illnesses and injury (Waldock et al. 2018).

Despite having a significantly higher increase in *T*_gi_, the older group felt slightly cooler throughout the trial and more comfortable at the end of the rest period, rating lower values than their younger counterparts. This was especially evident for the period when applying the absorbent pads and stretch clothing to the skin, as the younger group reported a significant rise in thermal sensation and became more uncomfortable, whereas the older group did not. The inability to report a change in thermal sensation and comfort when adding a layer of clothing highlights the vulnerability of older individuals in warm conditions and supports the previous evidence of a reduced whole body thermal sensitivity (Natsume et al. 1992; Taylor et al. 1995; Tochihara et al. 2011; Takeda et al. 2016) and thermal comfort (Natsume et al. 1992; Taylor et al. 1995; Waldock et al. 2018) in the aged. The combination of impaired autonomic and behavioural responses further increases the susceptibility of the older population and is a cause for concern in the current climate (Kenney et al. 2014; Waldock et al. 2018).

### Limitations

The present study assessed age-related differences in RSR using a whole body mapping approach with technical absorbents in male individuals. Unlike sweat capsules, this technique does not allow continuous monitoring of RSR development; hence, no analysis of the dynamics of sweat generation is possible. This disadvantage was accepted given the goal of covering a large part of the body for measurement, which is complicated with capsules (Taylor and Machado-Moreira 2013). Also the requirement to test the body in two parts may increase variability and thus reduce statistical power. To minimise this impact, all RSRs were standardised against the mean GSL variation of each trial. As work rates to achieve the same fixed heat production (200 W m^−2^) were defined in preliminary tests, and metabolic rate was not measured during the exercise period of the main trials, this may have introduced some variation in the actual workloads. However, as the intensity of the exercise was light-to-moderate, substantial changes in mechanical efficiency (from the submaximal calculations to the main trial) were not expected throughout the exercise. Finally, as this study was conducted solely on male participants within a fixed environmental condition (32 °C/50% RH), generalisation of results to females and to other climate types may require careful consideration. Future research should aim to investigate age-related differences in RSR in females and within a wider range of ambient conditions.

### Application

The findings of the present study have several applications from both a health-based and practical view point. The observed age-related declines in subjective and objective responses in the heat put older individuals at an increased risk of illness, injury, and, in extreme cases, mortality. Therefore, it may be necessary to revisit safety guidelines for working, exercising, and rest in hot conditions in those over the age of 65 years. Alternatively, aiming to alleviate these declines could promote better health in older individuals. The sweat mapping data presented within this study can be useful for clothing design, whereby different areas could be targeted to increase sweat evaporation, enhance cooling, and improve comfort. Moreover, the design of healthcare products and appliances may be tailored to individual needs based on the RSR of older individuals. For example, hospital beds and chairs that patients spend a large amount of time lying or sitting on could be designed to reduce irritation or the development of pressure sores caused partly from sweat accumulation. Finally, the current data relate directly into the design of thermal/sweating manikins and modelling in thermal physiology, providing more realistic sweat distribution patterns for young and older individuals.

## Conclusion

The main findings of this study were that despite equal heat production, healthy older individuals had (1) significantly lower GSLs than the young group and (2) significantly lower RSR at almost all body regions during rest and at the hands, legs, ankles, and feet during exercise—accepting the hypothesis. This study also demonstrated that older individuals had a greater increase in *T*_gi_ and a higher *T*_sk_ at the leg regions (the latter consistent with lowered sweating at the legs), but they felt slightly cooler when compared to younger individuals working at the same fixed heat production. These findings support the evidence of age-related deterioration in both autonomic and subjective responses in the heat and highlight the legs as the most affected body region, consistent with existing models of the impact of ageing on thermoregulation.

## Electronic supplementary material

Below is the link to the electronic supplementary material.

Supplementary file1 (TIF 1279 kb)

Supplementary file2 (DOCX 28 kb)

Supplementary file3 (DOCX 27 kb)

## Abbreviations

BSA: Body surface area
GSL: Gross sweat loss
RER: Respiratory exchange ratio
RH: Relative humidity
RPE: Rating of perceived exertion
RSR: Regional sweat rate
*T*_core_: Core temperature
*T*_gi_: Gastrointestinal (core) temperature
*T*_sk_: Skin temperature
