# Supplementary figures and images for: Body mapping of regional sweat distribution in young and older males
Source: Eur J Appl Physiol. 2020 Sep 29;121(1):109–25. doi: 10.1007/s00421-020-04503-5 (PMC7815578; doi:10.1007/s00421-020-04503-5)

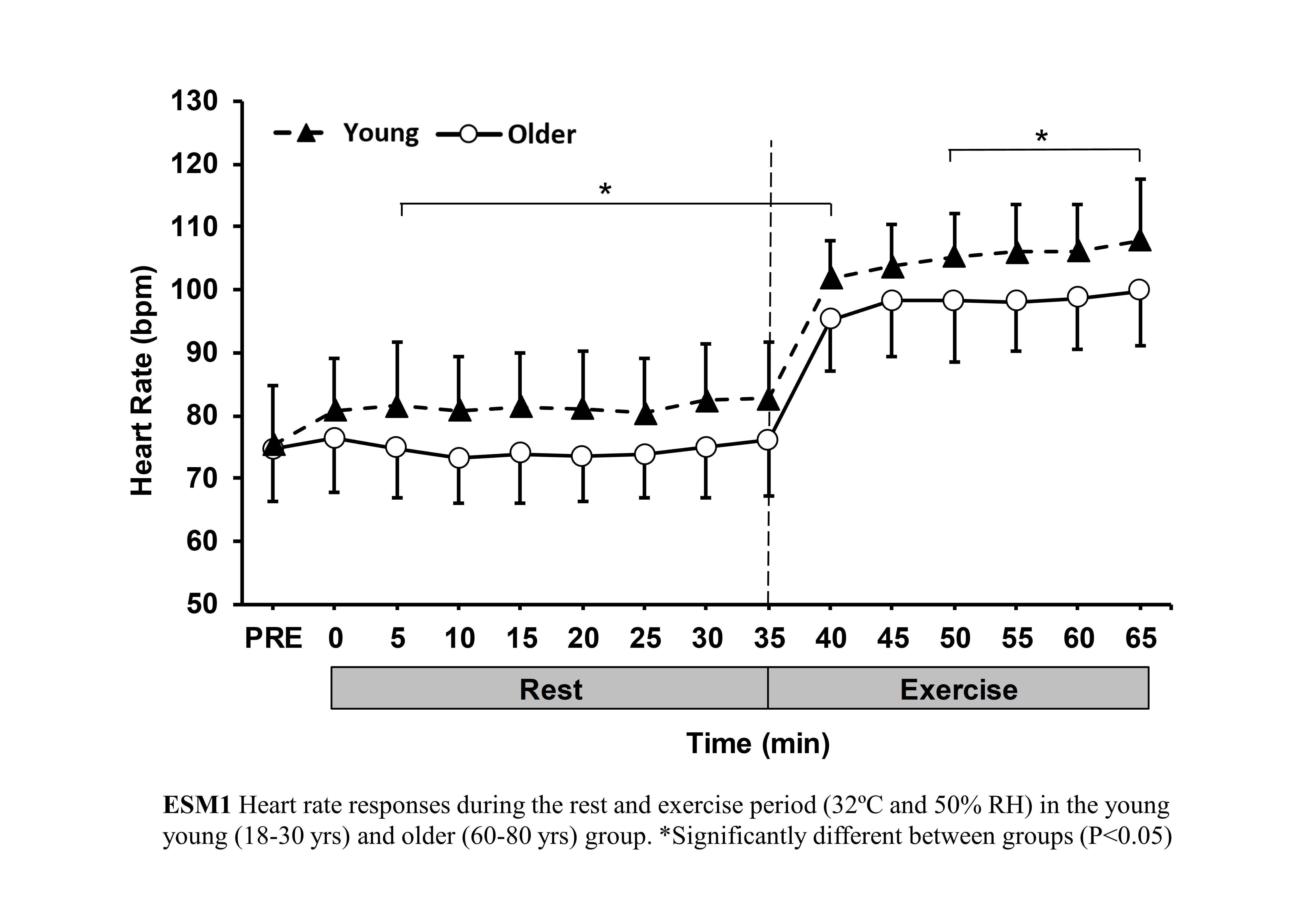

Supplement: Supplementary file 1 — Supplementary file1 (TIF 1279 kb) [file 421_2020_4503_MOESM1_ESM.tif]
