# Supplementary material for: Body mapping of regional sweat distribution in young and older males
Source: Eur J Appl Physiol. 2020 Sep 29;121(1):109–25. doi: 10.1007/s00421-020-04503-5 (PMC7815578; doi:10.1007/s00421-020-04503-5)
Supplement: Supplementary file 2 — Supplementary file2 (DOCX 28 kb) [file 421_2020_4503_MOESM2_ESM.docx]

| **Table A** | **Absolute Sweat Rates during rest period (g.m^-2.^h^-1^)** | | | | | | | | | | **Significance level** | |
| --- | --- | --- | --- | --- | --- | --- | --- | --- | --- | --- | --- | --- |
|  | **Young** | | | | | **Older** | | | | | **between age groups** | |
|  | **Min** | **Max** | **Median** | **Mean** | **SD** | **Min** | **Max** | **Median** | **Mean** | **SD** | **Absolute** | **Normalised** |
| Shoulders | 52.5 | 196.1 | 123.5 | 124.1 | 51.7 | 38.8 | 135.1 | 80.0 | 80.3 | 30.5 | * | - |
| Lateral upper chest | 49.8 | 200.0 | 105.2 | 106.7 | 37.7 | 20.3 | 145.7 | 78.6 | 70.2 | 38.7 | * | - |
| Medial upper chest | 40.4 | 166.9 | 107.4 | 101.7 | 38.0 | 12.8 | 139.8 | 54.2 | 62.7 | 44.4 | * | - |
| Lateral mid anterior torso | 38.3 | 230.8 | 113.5 | 118.9 | 46.5 | 4.6 | 225.1 | 64.6 | 77.0 | 56.6 | * | - |
| Medial mid anterior torso | 48.0 | 230.8 | 118.6 | 117.9 | 48.3 | 8.3 | 200.7 | 45.9 | 62.5 | 53.3 | ** | - |
| Sides | 70.1 | 168.6 | 108.6 | 112.3 | 30.7 | 14.7 | 241.0 | 62.5 | 79.1 | 60.2 | - | - |
| Lower anterior torso | 49.0 | 172.6 | 123.9 | 122.1 | 42.0 | 8.8 | 288.0 | 63.2 | 77.9 | 73.8 | - | - |
| Lateral posterior upper torso | 53.1 | 332.3 | 161.1 | 161.2 | 72.4 | 9.3 | 270.9 | 94.9 | 108.0 | 80.0 | - | - |
| Medial posterior upper torso | 89.8 | 432.3 | 216.9 | 218.4 | 87.3 | 20.7 | 332.1 | 97.8 | 123.0 | 89.1 | ** | - |
| Lateral posterior mid upper | 49.8 | 207.6 | 131.6 | 123.8 | 48.2 | 8.2 | 145.4 | 47.6 | 66.9 | 49.9 | ** | - |
| Lateral posterior mid lower | 65.1 | 188.1 | 127.7 | 126.1 | 38.8 | 20.7 | 266.8 | 57.2 | 81.4 | 68.1 | * | - |
| Centre posterior mid | 113.2 | 343.8 | 175.2 | 187.3 | 66.4 | 35.5 | 298.8 | 80.1 | 111.2 | 79.2 | * | - |
| Posterior lower torso | 83.8 | 275.1 | 145.8 | 151.8 | 53.1 | 12.9 | 293.0 | 52.7 | 83.4 | 77.5 | * | - |
| Anterior upper leg | 35.9 | 167.5 | 118.4 | 110.3 | 35.5 | 20.1 | 98.7 | 41.9 | 47.9 | 22.6 | ***# | * |
| Medial upper leg | 40.9 | 152.0 | 90.3 | 95.0 | 29.1 | 22.5 | 72.9 | 40.6 | 42.7 | 15.3 | ***# | - |
| Posterior upper leg | 25.8 | 160.5 | 82.2 | 87.0 | 37.3 | 12.6 | 54.5 | 43.7 | 36.4 | 15.1 | ***# | ** |
| Lateral upper leg | 26.6 | 216.0 | 104.2 | 106.1 | 51.1 | 12.8 | 70.0 | 42.6 | 40.7 | 16.9 | ***# | * |
| Lateral lower leg | 26.6 | 216.0 | 104.2 | 106.1 | 51.1 | 12.8 | 70.0 | 42.6 | 40.7 | 16.9 | ***# | - |
| Medial lower leg | 25.8 | 216.1 | 111.1 | 112.2 | 52.1 | 14.5 | 78.3 | 49.4 | 46.1 | 18.7 | ***# | - |
| Posterior lower leg | 25.8 | 160.5 | 82.2 | 87.0 | 37.3 | 12.6 | 54.5 | 43.7 | 36.4 | 15.1 | ***# | - |
| Anterior upper arm | 23.9 | 97.1 | 62.1 | 60.8 | 23.5 | 19.8 | 99.5 | 46.1 | 53.6 | 28.2 | - | * |
| Posterior upper arm | 23.6 | 107.8 | 58.3 | 58.5 | 27.0 | 4.8 | 102.9 | 51.6 | 55.5 | 28.7 | - | ** |
| Anterior lower arm | 20.2 | 118.6 | 67.1 | 72.0 | 26.1 | 10.7 | 126.2 | 50.2 | 59.9 | 37.1 | - |  |
| Posterior lower arm | 24.3 | 127.0 | 75.8 | 77.3 | 30.1 | 6.1 | 145.6 | 72.2 | 71.4 | 39.4 | - | * |
| Medial ankle | 66.5 | 256.6 | 145.2 | 146.9 | 50.4 | 28.4 | 136.9 | 70.5 | 73.1 | 30.7 | ***# | - |
| Lateral ankle | 74.6 | 235.0 | 129.9 | 131.5 | 42.3 | 23.7 | 101.6 | 63.2 | 60.9 | 23.3 | ***# | - |
| Hands | 66.7 | 157.2 | 98.9 | 102.6 | 25.1 | 40.8 | 177.4 | 77.5 | 92.4 | 41.5 | - | ***# |
| Feet | 45.5 | 102.5 | 72.2 | 72.2 | 18.0 | 18.5 | 58.8 | 38.7 | 38.8 | 12.3 | ***# | - |

**ESM2.** Sweat rate descriptive statistics for all regions during rest (Table A) and exercise (Table B) in young and older individuals. Significance level between age groups are displayed as **p* < 0.05, ***p* < 0.01, ****p* < 0.001 (young always higher). #Significant difference after Bonferroni correction.

| **Table B** | **Absolute Sweat Rates during exercise period (g.m^-2.^h^-1^)** | | | | | | | | | | **Significance level** | |
| --- | --- | --- | --- | --- | --- | --- | --- | --- | --- | --- | --- | --- |
|  | **Young** | | | | | **Older** | | | | | **between age groups** | |
|  | **Min** | **Max** | **Median** | **Mean** | **SD** | **Min** | **Max** | **Median** | **Mean** | **SD** | **Absolute** | **Normalised** |
| Shoulders | 168.7 | 539.9 | 315.8 | 333.5 | 107.9 | 119.7 | 484.0 | 332.3 | 325.1 | 104.2 | - | - |
| Lateral upper chest | 150.0 | 375.3 | 247.8 | 255.7 | 71.4 | 125.1 | 600.4 | 297.4 | 316.2 | 122.1 | - | ** |
| Medial upper chest | 90.1 | 568.1 | 242.1 | 272.8 | 134.8 | 119.7 | 501.0 | 292.4 | 290.4 | 119.7 | - | - |
| Lateral mid anterior torso | 121.5 | 466.1 | 252.7 | 273.6 | 85.4 | 97.5 | 643.5 | 273.7 | 304.9 | 162.7 | - | - |
| Medial mid anterior torso | 150.4 | 663.1 | 264.5 | 292.5 | 135.2 | 84.4 | 499.6 | 259.6 | 268.5 | 117.7 | - | - |
| Sides | 136.4 | 471.2 | 260.5 | 265.4 | 81.8 | 33.0 | 615.5 | 277.4 | 296.0 | 147.6 | - | - |
| Lower anterior torso | 97.7 | 320.4 | 190.6 | 199.0 | 57.3 | 113.2 | 625.6 | 230.0 | 270.0 | 133.6 | - | **# |
| Lateral posterior upper torso | 193.7 | 810.8 | 560.0 | 532.2 | 191.5 | 49.9 | 767.0 | 425.6 | 433.2 | 207.6 | - | - |
| Medial posterior upper torso | 251.0 | 1533.9 | 613.5 | 629.0 | 312.4 | 143.2 | 828.1 | 515.6 | 525.6 | 193.3 | - | - |
| Lateral posterior mid upper | 201.5 | 520.9 | 297.6 | 338.9 | 87.7 | 109.1 | 549.6 | 349.3 | 339.5 | 131.3 | - | - |
| Lateral posterior mid lower | 142.4 | 642.9 | 332.5 | 349.2 | 135.2 | 43.5 | 755.8 | 297.1 | 351.5 | 189.3 | - | - |
| Centre posterior mid | 138.1 | 1055.2 | 439.1 | 484.2 | 255.2 | 135.4 | 849.2 | 390.3 | 437.9 | 201.7 | - | - |
| Posterior lower torso | 153.9 | 840.6 | 378.1 | 437.6 | 232.5 | 95.7 | 593.9 | 360.5 | 353.6 | 161.1 | - | - |
| Anterior upper leg | 125.9 | 331.1 | 232.4 | 234.6 | 57.4 | 93.7 | 333.8 | 157.2 | 163.6 | 57.5 | ** | - |
| Medial upper leg | 150.3 | 296.5 | 207.2 | 209.8 | 37.5 | 60.9 | 244.0 | 159.9 | 163.7 | 59.7 | * | - |
| Posterior upper leg | 128.2 | 275.1 | 192.2 | 193.9 | 37.9 | 63.0 | 245.7 | 148.0 | 145.9 | 50.4 | ** | - |
| Lateral upper leg | 134.9 | 338.5 | 206.9 | 215.8 | 54.5 | 78.4 | 247.0 | 148.2 | 152.6 | 47.7 | ** | * |
| Lateral lower leg | 157.8 | 441.9 | 306.0 | 310.9 | 71.3 | 25.8 | 392.3 | 225.6 | 211.1 | 89.7 | ** | * |
| Medial lower leg | 184.7 | 603.7 | 333.0 | 338.6 | 103.3 | 60.0 | 379.9 | 199.4 | 217.0 | 89.1 | ** | * |
| Posterior lower leg | 138.3 | 416.3 | 247.2 | 243.2 | 63.7 | 56.4 | 239.6 | 174.5 | 168.6 | 53.7 | ** | - |
| Anterior upper arm | 95.6 | 241.2 | 172.5 | 166.3 | 51.2 | 110.2 | 292.0 | 178.6 | 182.3 | 46.4 | - | * |
| Posterior upper arm | 95.8 | 277.5 | 187.7 | 180.0 | 52.6 | 74.1 | 342.5 | 177.8 | 201.6 | 70.5 | - | ** |
| Anterior lower arm | 130.8 | 347.5 | 181.5 | 217.2 | 77.1 | 85.1 | 584.5 | 218.6 | 252.9 | 124.6 | - | * |
| Posterior lower arm | 137.7 | 316.9 | 229.5 | 239.6 | 60.9 | 93.6 | 540.5 | 238.2 | 255.2 | 116.1 | - | - |
| Medial ankle | 189.6 | 578.5 | 333.7 | 328.7 | 93.6 | 54.9 | 467.5 | 194.3 | 223.6 | 133.4 | * | - |
| Lateral ankle | 154.1 | 434.9 | 275.3 | 282.1 | 85.4 | 63.2 | 326.3 | 153.5 | 152.7 | 80.8 | ***# | ** |
| Hands | 120.3 | 311.5 | 210.6 | 215.0 | 55.4 | 79.2 | 259.1 | 152.6 | 161.3 | 64.5 | * | - |
| Feet | 69.6 | 181.8 | 136.9 | 135.3 | 28.2 | 47.2 | 155.9 | 83.5 | 89.6 | 29.7 | ***# | * |
