# Supplementary material for: Body mapping of regional sweat distribution in young and older males
Source: Eur J Appl Physiol. 2020 Sep 29;121(1):109–25. doi: 10.1007/s00421-020-04503-5 (PMC7815578; doi:10.1007/s00421-020-04503-5)
Supplement: Supplementary file 3 — Supplementary file3 (DOCX 27 kb) [file 421_2020_4503_MOESM3_ESM.docx]

|  | **Regional Skin Temperature (°C) at each time point in the Young and Older group** | | | | | | | | | |
| --- | --- | --- | --- | --- | --- | --- | --- | --- | --- | --- |
|  | **Baseline** | | **Pre-Pad-Rest** | | **Post-Pad-Rest** | | **Pre-Pad-Ex** | | **Post-Pad-Ex** | |
|  | **Young** | **Older** | **Young** | **Older** | **Young** | **Older** | **Young** | **Older** | **Young** | **Older** |
| Anterior upper arm | 34.2 ± 0.7 | 34.0 ± 0.8 | 35.6 ± 0.6***# | 35.8 ± 0.6***# | 35.7 ± 0.8 | 35.8 ± 0.7 | 34.1 ± 0.7***# | 33.6 ± 0.9***# | 34.7 ± 0.7***# | 34.3 ± 0.9***# |
| Anterior lower arm | 34.3 ± 0.7 | 33.9 ± 0.9 | 35.5 ± 0.6***# | 35.7 ± 0.7***# | 35.8 ± 0.5 | 35.9 ± 0.8 | 33.8 ± 0.6***# | 33.6 ± 0.9***# | 34.5 ± 0.5***# | 34.3 ± 0.7***# |
| Posterior upper arm | 32.4 ± 0.9 | 32.4 ± 0.4 | 34.4 ± 0.7***# | 34.6 ± 0.4***# | 34.9 ± 0.7***# | 35.0 ± 0.7***# | 34.7 ± 0.8 | **33.9 ± 0.9***#** | 35.4 ± 0.7***# | **34.6 ± 1.1***#** |
| Posterior lower arm | 34.0 ± 0.7 | 33.7 ± 0.8 | 35.2 ± 0.4***# | 35.2 ± 0.6***# | 35.6 ± 0.6***# | 35.7 ± 0.7***# | 34.3 ± 0.7***# | 34.3 ± 1.1***# | 35.0 ± 0.6***# | 35.0 ± 1.1***# |
| Upper torso | 34.8 ± 0.9 | 34.3 ± 0.7 | 35.7 ± 0.8***# | 36.1 ± 0.6***# | 35.7 ± 0.7 | 35.8 ± 0.8 | 34.1 ± 0.9***# | 33.8 ± 1.0***# | 34.8 ± 0.6***# | 34.6 ± 0.8***# |
| Mid torso | 34.9 ± 0.8 | **34.1 ± 0.8** | 35.4 ± 1.0* | 35.5 ± 0.8***# | 35.5 ± 0.8 | 35.6 ± 0.8 | 33.8 ± 0.9***# | 33.5 ± 1.0***# | 34.7 ± 0.5***# | 34.3 ± 0.9***# |
| Lower torso | 34.5 ± 1.0 | **33.1 ± 1.2** | 35.3 ± 0.9** | 35.2 ± 0.8***# | 35.4 ± 0.8 | 35.2 ± 0.8 | 34.0 ± 0.8***# | 33.4 ± 1.0***# | 34.5 ± 0.6***# | 34.1 ± 0.8***# |
| Upper back | 34.7 ± 1.0 | 34.3 ± 0.7 | 35.0 ± 1.0 | 35.6 ± 0.9***# | 35.3 ± 0.8 | 35.7 ± 1.0 | 33.7 ± 0.9***# | 33.6 ± 1.1***# | 35.2 ± 0.6***# | 35.0 ± 0.9***# |
| Mid back | 34.0 ± 1.0 | 34.1 ± 0.7 | 34.6 ± 0.9** | 35.2 ± 0.8***# | 34.8 ± 0.8 | 35.2 ± 0.9 | 33.8 ± 0.7** | 33.3 ± 1.2***# | 35.0 ± 0.6***# | 34.5 ± 1.0***# |
| Lower back | 34.1 ± 1.0 | 33.7 ± 0.7 | 34.6 ± 0.7* | 34.8 ± 0.8***# | 34.5 ± 0.7 | 34.6 ± 0.9* | 34.0 ± 0.8* | **33.3 ± 1.1***#** | 35.1 ± 0.6***# | 34.3 ± 0.9***# |
| Anterior upper leg | 32.4 ± 1.0 | 32.6 ± 1.0 | 34.0 ± 0.8***# | **34.7 ± 0.9***#** | 34.3 ± 0.8* | 34.8 ± 0.7 | 33.9 ± 1.0* | 34.5 ± 1.1 | 34.3 ± 1.0** | 34.9 ± 1.4***# |
| Anterior lower leg | 32.8 ± 0.6 | **33.4 ± 0.7** | 33.3 ± 0.7** | **34.4 ± 0.9***#** | 33.7 ± 0.7***# | **34.8 ± 0.8***#** | 34.3 ± 0.5** | **35.7 ± 1.3**** | 34.5 ± 0.5***# | **35.9 ± 1.4***#** |
| Posterior upper leg | 32.8 ± 0.8 | 33.1 ± 0.9 | 33.7 ± 0.7***# | **34.5 ± 0.8***#** | 34.1 ± 0.6***# | **34.9 ± 0.7***#** | 34.5 ± 0.7** | **35.4 ± 1.0*** | 34.8 ± 0.7** | **35.7 ± 1.2** |
| Posterior lower leg | 32.7 ± 0.6 | 33.2 ± 1.0 | 33.4 ± 0.6** | **34.4 ± 0.8***#** | 33.8 ± 0.6* | **34.6 ± 0.8*** | 34.6 ± 0.4***# | **35.9 ± 1.4***#** | 34.8 ± 0.5** | **36.0 ± 1.4** |
| Hand | 34.1 ± 1.7 | **32.3 ± 1.9** | 35.7 ± 0.4** | 35.7 ± 1.0***# | 35.9 ± 0.7 | 35.6 ± 1.2 | 35.4 ± 0.6* | 35.4 ± 1.3 | 35.7 ± 0.3** | 35.9 ± 1.1***# |
| Foot | 31.3 ± 2.1 | 30.2 ± 2.5 | 33.1 ± 2.0***# | 32.0 ± 1.7***# | 33.4 ± 1.6 | 32.5 ± 1.5 | 34.8 ± 1.3* | 35.6 ± 2.0***# | 35.2 ± 1.0 | 35.7 ± 1.8 |
| Mean of all regions | 33.6 ± 1.0 | 33.3 ± 1.0 | 34.6 ± 0.8 | 35.0 ± 0.8 | 34.9 ± 0.7 | 35.1 ± 0.9 | 34.2 ± 0.8 | 34.3 ±1.1 | 34.9 ± 0.6 | 34.9 ± 1.1 |

**ESM3.** Mean ± SD of regional skin temperature (°C) at each time point in the young and older age group. Significantly different from previous time point: displayed as **p* < 0.05, ***p* < 0.01, ****p* < 0.001 (always significant increase unless highlighted grey). #Significant difference after Bonferroni correction. **Bold** **text** indicates significant difference from younger age group.

= significant decrease from previous time point.
